# Autonomy in the context of cognitive demands—is the resource becoming a stressor?

**DOI:** 10.1007/s00420-023-01966-9

**Published:** 2023-03-17

**Authors:** Sabrina Zolg, Britta Herbig

**Affiliations:** Institute and Clinic for Occupational, Social and Environmental Medicine, University Hospital, LMU Munich, Ziemsensstr.5, 80336 Munich, Germany

**Keywords:** Digitalization, Autonomy, Cognitive demands

## Abstract

**Objective:**

Autonomy is often associated with positive linear effects on health whereas non-linear correlations have received only sporadic attention. Assuming that the use of autonomy also represents a cognitive demand, this study examines whether health effects of autonomy change depending on further cognitive demands and whether curvilinear relationships can be identified.

**Methods:**

A survey was carried out in three SMEs with established work analysis questionnaires. 197 Employees were classified into groups with high and with low cognitive demands by means of a two-step cluster analysis. This was modeled as moderator together with curvilinear effects of autonomy in regression analyses.

**Results:**

Curvilinear associations were found for emotional exhaustion, cynicism, and anxiety. They were strongest for anxiety. No moderating effects of cognitive demands and no consistently significant modeled relations were found.

**Conclusion:**

The results confirm that autonomy has a positive influence on the health of employees. However, autonomy should not be seen as an isolated resource but embedded in the organizational and societal context.

## Introduction

In essence, autonomy at work, including some conceptual gradations such as degree of freedom, latitude or control, is to be understood as the degree of influence on the processes and results of one's own work (Sparks et al. 2001; Morgeson and Humphrey 2006; Sichler 2006). Influence can refer to the following three areas: first, the organization of action, second, the design of ways of working, and third, the ability to make decisions and set tasks oneself (Ulich 2011). In a similar way, Humphrey et al. (2007) describe three facets of autonomy: work scheduling autonomy as the freedom to control the scheduling and timing of work, work methods autonomy as the freedom to control which methods and procedures are utilized, and decision-making autonomy as the freedom to make decisions at work. They also propose differences in the magnitude of relations of the facets to work outcomes. Although this differentiation in facets has already existed for a long time (Jackson et al. 1993; Karasek et al. 1998), autonomy or job control is often equated only with the last area of influence, that is, decision-making autonomy.

Autonomy in general is understood as a resource in theories of work psychology, such as the Job Characteristics Model (Hackman and Oldham 1976), the Job Demand Control Model (Karasek 1979; Karasek and Theorell 1990), or in its extension to the Job Demands Resources Model (Bakker and Demerouti 2007). Autonomy is thus not only one of the central components of work design that promotes learning, personality and health, but can furthermore be classified as a fundamental human need. That is, an increase in autonomy has a potentially health- and personality-promoting effect, while a low level of autonomy conversely has negative effects (for review, Bonde 2008; Stansfeld and Candy 2006; Spector 1986; Häusser et al. 2010; de Lange 2003; van der Doef and Maes 1999, Rau and Buyken 2015; Nixon et al. 2011; Theorell et al. 2015, Theorell et al. 2016). This effect has been found for numerous health, well-being, and performance parameters, such as job performance and work engagement (Schaufeli et al. 2009; Nagami et al. 2010), well-being and job satisfaction (Cheung et al. 2015), emotional exhaustion (Van Ruysseveldt et al. 2011), depression (Ahlin et al. 2018; Madsen et al. 2017), mental health (Bentley et al. 2015; Butterworth et al. 2011), psychiatric status (Stansfeld et al. 1999), and even somatic conditions such as high blood pressure (Steptoe and Willemsen 2004). In the modern highly connected world of work, demands, stressors and resources have changed significantly (Allvin et al. 2011; Höge 2011). In many cases, the new forms of work are associated with higher cognitive demands and increased task complexity in addition to work intensification (Burchell et al. 2002; Cascio and Montealegre 2016; Burke and Cooper 2002; Meyer and Hünefeld 2018; Schaufeli and Taris 2014).

At the same time, the degree of autonomy in work has increased for many employees, especially in the course of flexibilization (Wood 2011; Pongratz and Voß, 2003). Often, this is linked to new management methods that place a strong responsibility for task and goal fulfillment on individual employees (Moldaschl and Voß, 2002; Sichler 2006; Höge 2011; Bredehöft et al. 2015). Opportunities thus become necessities, requiring increased responsibility as well as higher performance (Shazad 2021; Nagel 2010). These factors, as well as new forms of management, some of which are manifested in higher participation opportunities for employees, can thereby provide for an increase in psychological stress (Busck et al. 2010). Under these conditions, autonomy can change from a resource to a stressor because an increase in autonomy in the new world of work is linked to demands for additional (self-)organization and planning, which ties up "psychological capital" such as self-efficacy or resilience (Shazad 2021; O’Donnell et al. 2015).

Similar considerations can be found in theoretical approaches, such as the vitamin model (Warr 1990), the assumption of a "too-much-of-a-good-thing" (TMGT) effect (Grant and Schwartz 2011; Pierce and Aguinis 2013), or a "choice overload" (Schwartz 2004), which assumes that too many fundamentally beneficial working conditions, such as autonomy, will eventually cause the positive effects to stagnate or even turn into negative effects.

The assumption of an inverted U-shaped or otherwise nonlinear effect of autonomy, job control or decision authority has been investigated by a number of studies in recent years. The health outcomes collected in these studies are as diverse as the occupational groups and topics of focus. Chung-Yan (2010) found a stagnation of the positive effect of autonomy on well-being and job satisfaction for employees with high job complexity, while employees with lower complexity continued to benefit positively linearly from autonomy. In contrast, De Jonge and Schaufeli (1998) found the highest values for emotional exhaustion at an intermediate level of autonomy and thus an inverted U-shaped relationship. Nonlinear correlations of autonomy with job satisfaction or well-being and emotional exhaustion were found in both Rydstedt et al. (2006) and Kubicek et al. (2014). Stiglbauer and Kovacs (2018) partially demonstrated that autonomy shows inverted U-shaped trajectories with well-being but that it is strongly dependent on the operationalization and the respective facet of autonomy. Flynn and James (2009) showed in their experimental study that cardiovascular reactivity was similar for high and low control. In addition to nonlinear trajectories, there are also results on negative linear relationships: Joensuu et al. (2010, 2012) showed that high rather than low decision latitude increased the risk of depression and cardiovascular and alcohol-induced disease. Signs of a higher risk of cardiovascular disease were also found by McCarthy et al. (2014) to be present in older workers with high job control. In an experimental design, O’Donnell et al. (2015) found that although no negative effects of autonomy were reported in the surveys, the physiological parameters collected showed that increased autonomy can induce stress responses. However, the results of these studies differ considerably, and there are some studies that could not find significant results despite the investigation/assumption of a negative linear or curvilinear relationship. Jeurissen and Nyklíček (2001) were also unable to confirm curvilinear relationships.

These ambiguous, inconsistent results suggest that other factors and constellations of demands might be responsible for the varying types of relationships between autonomy and health outcomes, and that further individual and contextual factors, potentially moderators (Stiglbauer 2017) and organizational demands (Clausen et al. 2022) should be taken into account, as is already evidenced in the Job Demand Control model where the combination of high job demands and low job control are usually associated with negative health effects (de Lange et al. 2002; van der Doef and Maes 1999). Thus, in addition to the degree of autonomy, the efficacy of positive effects also seems to depend on whether the resource autonomy can come into play in the concrete work condition structure (Stiglbauer 2017).

Against this background, we argue in the remainder of this paper that task-related requirements on cognition might pose one especially important moderator for the relationship between autonomy and health of employees in those working conditions that have changed as a result of developments in digitization, that is, increased work intensity and complexity, increased quantitative demands, and the cognitive demands on employees (Meyer and Hünefeld 2018; Zolg et al. 2021). The reasoning behind this assumption is that the use of autonomy and especially decision-making autonomy requires cognitive performance, like e.g. planning and complex problem-solving. If this requirement acts “on top” of already high task-related cognitive demands, it might be overtaxing and, therefore eliminate the positive effect on employees’ health. That is, we propose an interaction of autonomy and task-related cognitive demands. Dettmers and Bredehoeft (2020) use a similar reasoning in their study; however, instead of existing work design concepts they propose a new concept called “job design demands” as mediator between autonomy and emotional exhaustion and irritation, respectively, that can explain potential negative effects and is labeled the “dark side” of autonomy by the authors. In contrast, our approach is parsimonious and tries to retain the positive concept of autonomy including its requirements as an inherently health- and personality-promoting work characteristic (Hackman and Oldham 1976; Humphrey et al. 2007). Effects of different facets of autonomy could then be hypothesized as depending on the cognitive performance each facet requires and adds to other task-related cognitive demands. For example, decision-making autonomy as a complex problem-solving task might tie up more cognitive resources than activity latitude.

The aim of this paper is therefore to determine whether the influence of autonomy on various health factors depends on the level of task-related cognitive demands. We assume that autonomy should basically be evaluated as a resource, which, however, can develop into a stressor under the condition that task-related cognitive demands are already high and trying. Task-related demands are operationalized by a clustering of a number of different demands usually surveyed in work design questionnaires (see “Methods and Materials” section). We look at these proposed associations for a range of health outcomes to determine whether general patterns can be identified. We also expect different results for the three facets of autonomy. For example, as decision latitude is probably the facet with the highest cognitive requirements, we expect that we will see a moderation with task-related cognitive demands in the sense of curvilinear effects for participants with high task-related demands, whereas this might not be the case for activity or design latitudes.

The following hypotheses and research questions are examined:

1. Is there a positive effect of autonomy that can be confirmed consistently for all health parameters surveyed? (Hypothesis 1: positive linear association).
2. Is this positive effect of autonomy moderated by the level of task-related cognitive demands an employee has? (Hypothesis 2a: moderated association).
3. Are high task-related cognitive demands likely to cause the positive effect to stagnate or reverse at some point? (Hypothesis 2b: direction of moderation: low/middle cognitive demands = positive linear association; high cognitive demands = curvilinear/nonlinear association).
4. Do these effects differ for the three facets of autonomy (activity-, design-, and decision-latitude)?

## Methods and materials

### Design

We used a cross-sectional design to survey the latitudes at work, task-related cognitive demands and health outcomes of employees. The study is part of the LedivA project (Leistungsregulierung bei digital vernetzter Arbeit-Performance regulation in qualified digitally connected work) that investigates the working conditions and the physical and mental strain resulting from digitally connected work. All data were gathered between September 2019 and December 2019. The study was approved by the Ethics Committee at the Faculty of Medicine, Ludwigs-Maximilians-University, Munich (ID: 19–430).

### Participants

Employees of three small and medium-sized enterprises (SME) were recruited. Two of the SMEs were from the manufacturing sector and one was from the service sector. As employees at industrial workplaces would otherwise not be reached, the survey was conducted as a paper-and-pencil questionnaire and fill-in time was working time for all participants. Due to data protection measures, employees could not be directly approached but via contact persons in the enterprises who were informed in advance about the objectives and procedures of the project. Employees received this information via internal communications and in the informed consent forms that were sent together with the questionnaires as packages to the contact persons in all companies who distributed these to all employees at a fixed start date. A four-week deadline for submitting the questionnaires were communicated to everyone. Those who wanted to participate in the survey had to return the signed consent forms in separate envelopes. The questionnaires could be returned in sealed envelopes put in locked boxes (both provided by the project) in easily accessible rooms in the enterprises. As no direct contact with the employees was possible, contact persons received up to two reminders by email and telephone to be relayed to all employees. Of the 433 employees invited to take part in the survey, 197 (45.5%) completed and returned the questionnaire. The response rate in the companies was 31.8%, 42.6% and 100%, respectively. Although on the low side, this response rate falls within the range of average rates and continuing decline in response reported in literature for organizational research (Anseel et al. 2010; Baruch and Holtom 2008; Weigold et al. 2019). The range of response rates results from the respective organizational structures (e.g., number of sales representatives difficult to reach, availability of in-house mail, size of enterprise with the smallest enterprise showing full participation), and situations (e.g., holidays, volume of work in the respective survey timeframe with one enterprise having a strong seasonal business) as well as the different approaches to the survey of the responsible contacts and their motivating power.

### Measures

Descriptive values, internal consistencies and intercorrelations for all scales are presented in Table 1. Sample items from German questionnaires are in most parts ad hoc translations.

**Table 1:** Means, standard deviations, Cronbach’s alpha reliability coefficients, and intercorrelations between all variables

	Variables	M ± SD	Scale range (items)	1	2	3	4	5	6	7	8	9	10	11	12	13
1	Age	41,92 ± 12,59														
2	Gender		0 = *m*;1 = *f*	− 0.08												
3	Job description	1.97 ± 0.86	1–4^a^	0.08	− 0.31***											
4	Subscale activity latitude	3.86 ± 0.87	1–5^a^ (3)	− 0.04	0.05	0.11	(0.78)									
5	Subscale decision latitude	2.87 ± 0.91	1–5^a^ (3)	0.07	− 0.21***	0.38***	0.54***	(0.78)								
6	Subscale Design Latitude	3.17 ± 1.08	1–5^a^ (3)	0.08	− 0.25***	0.29***	0.53***	0.58***	(0.89)							
7	Overall autonomy	3.30 ± 0.80	1–5^a^ (9)	0.05	− 0.18*	0.31***	0.80***	0.84***	0.86***	(0.89)						
8	Emotional exhaustion	3.30 ± 1.20	1–6^a^ (3)	0.06	0.02	0.08	− 0.12	− 0.19***	− 0.14*	− 0.19*	(0.92)					
9	Cynicism	2.78 ± 1.20	1–6^a^ (3)	− 0.09	− 0.04	0.04	− 0.17*	− 0.18*	− 0.14*	− 0.19***	0.56***	(0.88)				
10	Anxiety	2.40 ± 1.18	1–7^a^ (7)	− 0.04	0.28***	− 0.15*	− 0.20***	− 0.33***	− 0.27***	− 0.32***	0.49***	0.43***	(0.83)			
11	Cognitive irritation	3.61 ± 1.60	1–7^a^ (5)	0.16*	− 0.12	0.24***	− 0.11	0.02	0.00	− 0.04	0.58***	0.33***	0.37***	(0.88)		
12	Workability index	4.03 ± 0.67	1–5^a^ (3)	− 0.03	− 0.04	0.13	0.26***	0.23***	− 0.27***	0.30***	− 0.50***	− 0.47***	− 0.41***	− 0.34***		
13	Well-being	2.84 ± 1.16	1–5^a^ (5)	0.13	− 0.10	0.11	0.15*	0.23***	0.29***	0.28***	− 0.43***	− 0.42***	− 0.49***	− 0.32***	.51***	(0.90)
14	Cognitive demands cluster		0 = middle/ low; 1 = high	0.03	− 0.31***	0.43***	0.26***	0.35***	0.37***	0.39***	0.11	− 0.00	− 0.17*	0.21***	0.08	0.16*

Correlation coefficients according to Spearman (Cognitive Demands/Job Description), Kendall (Job Description—Gender) and Pearson (all other variables), reliability according to Cronbach's Alpha (in parentheses), *p value ≤ .05, **p value ≤ .01, ***p value ≤ .001.

^a^Higher values = higher expression

#### a) Dimensions of autonomy

Autonomy was assessed with the German self-report instrument "Activity and Work Analysis" (TAA) by Glaser et al. (2020) containing three subscales: *activity latitude* (e.g., I can decide for myself which working methods and tools to use, 3 items), *design latitude* (e.g., My work permits using my own ideas, 3 items), and *decision latitude* (e.g., I can make my own decisions about work goals, 3 items). All three subscales use a 5-point Likert-scale (1 = no, not at all, 5 = yes, exactly). *Overall task latitude* (autonomy) contains all nine items.

#### b) Task-related cognitive demands

*Mental demands* were assessed with four items from the TAA (Glaser et al. 2020) (e.g., My work requires weighing various aspects to complete my tasks (1 = no, not at all, 5 = yes, exactly)). *Knowledge demands* were measured with a single item (Does your work require broad knowledge?) from the Copenhagen Psychosocial Questionnaire (COPSOQ, Kristensen et al. 2005; German version Nübling et al. 2005) with a 5-point Likert scale from 1 = to a very small extent to 5 = to a very large extent.

Further cognitive demands were assessed with the German version (Stegmann et al. 2019) of the Work Design Questionnaire (WDQ, Morgeson and Humphrey 2006). With four items each and 5-point Likert-type scales, these include *skill variety* (e.g., The job requires a variety of skills (1 = strongly disagree, 5 = strongly agree), *complexity* (e.g., The job requires that I only do one task or activity at a time (1 = strongly disagree; 5 = strongly agree; recoded), *problem-solving* (e.g., The job often involves dealing with problems that I have not met before), and *information processing* (e.g., The job requires me to monitor a great deal of information).

#### c) Health outcomes

To cover a range of possible health outcomes, we collected short- and long-term general and work-related variables. We assessed *anxiety* in a nonclinical context (e.g., I avoid addressing my supervisor at work; 7 items) with a 7-point Likert-type scale (1 = strongly disagree; 7 = strongly agree) from Mohr and Müller (2014).

The German version of the Maslach Burnout Inventory (Maslach and Jackson 1981; Büssing and Perrar 1992) was used to assess the two key burnout components *emotional exhaustion* (e.g., At the end of a workday, I feel used up, 3 items) and *cynicism* (e.g., I have become more cynical about whether I am making any contribution with my work, 3 items) with a 6-point frequency scale (1 = never; 6 = very often).

*Well-being/depression* was assessed with the WHO Five Well-Being Index (World Health Organization (WHO) 1998) with five items (e.g., Over the last two weeks, I wake up feeling fresh and rested) using a 6-point scale (0 = at no time; 5 = all of the time).

To assess *cognitive irritation* (e.g., I have difficulty relaxing after work), we used a 7-point Likert-type scale (1 = strongly disagree; 7 = strongly agree) (Mohr et al. 2005).

To assess work ability, we used an abbreviated German version of the WAI Workability Index developed by Tuomi et al. (1998) (Müller et al. 2016). The items (work ability in relation to physical and mental demands of work) used a 5-point Likert scale (1 = very poor; 5 = very good) and an overall ability rating with an 11-point scale (0 = cannot work at all; 10 = currently the best work ability).

#### d) Control variables

Information on control variables *age* and *gender* are based on self-reported data from the questionnaire. Participants were also required to match themselves to a *job description* appropriate for their position. They could choose between four different types of jobs: semiskilled/assisting work, qualified work, work with specialist responsibility, or work with extensive management responsibilities and decision-making powers.

### Statistical analyses

We conducted descriptive analyses for each of the outcome and latitude variables by using means and standard deviations (SDs), frequencies, and percentages. Furthermore, to capture the relationships of the variables, we performed correlation analyses.

To identify the employees with different levels of cognitive demands, we used a two-step cluster analysis (e.g., Benassi et al 2020) with the six measured task-related cognitive demands. The log-likelihood method was used for distance measures. The number of clusters was not prespecified. As an overall goodness-of-fit measure of the cluster structure the silhouette measure of cohesion and separation (Kaufman and Rousseeuw 1990; Rousseeuw 1987) was used. The silhouette value essentially captures how similar an object is to its own cluster (cohesion) compared to other clusters (separation); the coefficient is basically the difference between cluster separation and cohesion divided by the maximum of the two (Rousseeuw 1987). It ranges from − 1 to 1. A score above 0 ensures that the within-cluster distance and the between-cluster distance is valid; scores above 0.2 are usually evaluated as fair, at or above 0.5 as good (Tkaczynski 2017). To determine the best cluster solution, that is, the most parsimonious cluster solution with the best fit, we adopted the procedure used by Benassi et al. (2020) with evaluations for up to four clusters as a reasonable number for classification. Akaikes Information Criterion (AIC) and Bayesian Information Criterion (BIC) changes were calculated as the difference between two cluster solutions starting from the most parsimonious (one cluster) to the least parsimonious (four clusters). With this procedure, a two-cluster solution proofed to be the best solution. From this, we derived the binary variable *cognitive demands* (CD) (“low/medium cognitive demands” = 0, “high cognitive demands” = 1). For the results of the cluster analysis, see "Results of regression analyses".

In preparation for the subsequent analyses, we identified unusual cases by specifically looking for deviations and anomalies from the normal values of the groups. Two cases from the cluster with higher cognitive demands were then excluded from the analysis. To assess whether cognitive demands have a moderating curvilinear effect on the relationship between autonomy and various health outcomes, we then conducted hierarchical moderated regression analyses.

In step one, we controlled for age. To avoid multicollinearity problems, we decided against using gender as a control variable because chi-square tests confirmed that gender is already represented in the clusters; that is, in the cluster of high cognitive demands, 70.1% of participants are male (see section "Cluster analysis"). We also decided against including the variable job description as a control variable for similar reasons. As seen in the results in the correlation table, correlations are clearly visible between this variable and the variables age and gender.

In a second step, we added the variables on autonomy (A), the cluster variable (CD) and the interaction term to test the main effects and the linear moderation.

In steps three and four, quadratic and cubic terms for autonomy and their respective interaction with the cognitive demands were added to test for curvilinear moderation effects.

All analyses were conducted with IBM SPSS Statistics 26.

## Results

### Study population

A total of 193 employees (37.1% women, 60.9% men) with an average age of 41.9 years (19–70, SD = 12.6) were included in the analysis. A secondary school diploma or lower was held by 34.4%, an intermediate school-leaving certificate by 36% and an applied or general university entrance qualification by 28.9%. Based on data from the companies, 83.2% of the participants worked in the production sector and 16.8% worked in the service sector. Job descriptions of the employees covered semiskilled/assisting work (20.0%) and qualified work (55.6%) as well as independent work with specialist responsibility (20.6%) and work with extensive management responsibilities and decision-making powers (3.9%). Duration of employment at the respective company was rather high: Only 6.8% of participants worked for less than one year at their job, whereas 27.4% worked more than five years and 33.7% more than ten years at their current job.

### Cluster analysis

**Fig. 1:**
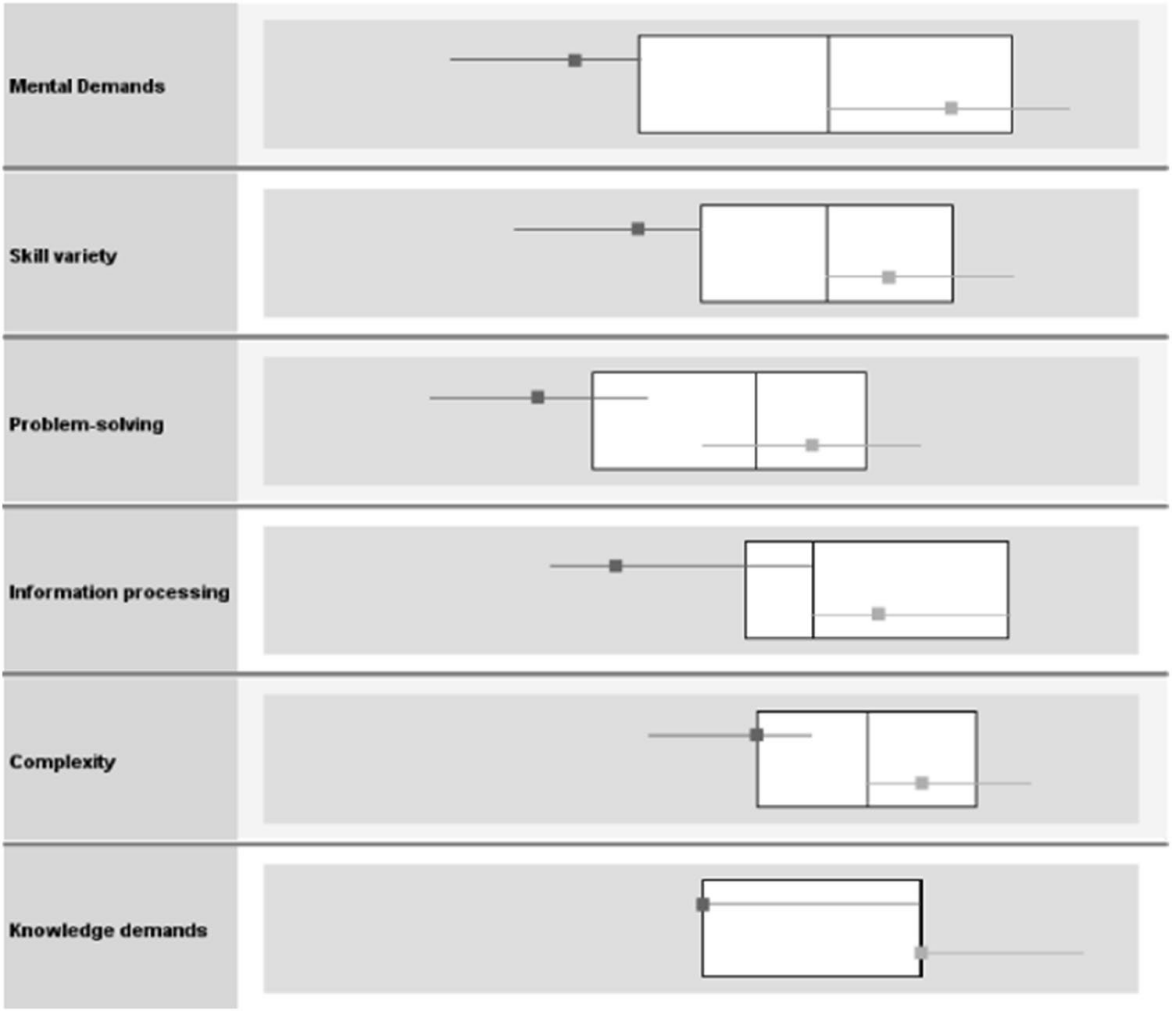
Boxplots of value distributions in the general sample (median, 25th and 75th quantile), the low/medium cognitive demands group (upper dark grey line) and the high cognitive demands group (lower light grey line)

The two-step cluster analysis of the six task-related cognitive demands resulted in a two-cluster solution. Changes in AIC and BIC confirmed this solution as the best, most parsimonious model (∆ AIC: 2 vs. 1 cluster = − 206.33, 3 vs. 2 = − 65.90, 4 vs. 3 = − 19,19; ∆ BIC: 2 vs. 1 cluster = − 167.18, 3 vs. 2 = − 26.74, 4 vs. 3 = 19.96). The two cluster outcome showed a fair silhouette measure of cohesion and separation of 0.50 indicating a medium-sized structuring, while with fixed cluster numbers the silhouette measures were considerably lower (3 and 4 clusters 0.30). Therefore, the two-cluster solution was used in all further analyses. The two groups could be defined as one group with high cognitive demands (*n* = 134) and one group with low/medium cognitive demands (*n* = 59). The means of the six included variables dividing the cluster differed by an average of 1.15 points on the 5-point scales; the predictor importance for each variable for the clustering shows a rather high relative importance of all variables with mental demands being the most and knowledge demands being the least discriminative between groups (see Table 2 and Fig. 1).

**Table 2:** Characteristics of the Cluster “cognitive demands”

Cluster “cognitive demands”	Mental demands	Skill variety	Complexity	Information processing	Problem-solving	Knowledge demands
	M ± SD	M ± SD	M ± SD	M ± SD	M ± SD	M ± SD
Low/medium	2.71 ± 0.62	2.84 ± 0.61	3.13 ± 0.76	3.17 ± 0.67	2.21 ± 0.72	3.03 ± 0.83
High	4.15 ± 0.61	4.07 ± 0.60	4.06 ± 0.61	4.15 ± 0.57	3.54 ± 0.73	4.05 ± 0.69
Predictor importance	1.0	0.83	0.47	0.59	0.70	0.46

The (clusters of) cognitive demands also differ in their composition regarding age, gender, and job description. More than half of all women are found in the group with lower cognitive demands, while more than two-thirds of all men are found in the cluster with high cognitive demands. In both clusters, approximately half of the employees perform qualified work. Work with extensive management responsibilities and decision-making powers is not present in the cluster with lower cognitive demands (see Table 3). With regard to the age distribution, the significant difference between the clusters can be attributed exclusively to the high proportion of trainees in the cluster with lower cognitive demands.

**Table 3:** Cognitive demands cluster and sample descriptives

	“High cognitive demands” (*n* = 132)	“Low/medium cognitive demands” (*n* = 59)	Chi-square test
Sex			
Male	93 (70.5%)	22 (37.3%)	*p* < .001
Female	37 (28.0%)	35 (59.3%)	
Missing	2 (1.5%)	2 (3.4%)	
Job description			
Semiskilled/assisting	14 (10.6%)	23 (39.0%)	*p* < .001
Qualified work	73 (55.3%)	29 (49.2%)	
Work with specialist responsibility	35 (26.5%)	2 (3.4%)	
Work with extensive management responsibilities and decision-making powers	7 (5.3%)	0 (0.0%)	
Missing	3 (2,8%)	5 (8.5%)	
Age			
< 25	10 (7.6%)	10 (17.0%)	*p* < .001
26–45	61 (46.2%)	22 (37.3%)	
46–70	60 (45.5%)	25 (42.4%)	
Missing	1 (0.8%)	2 (3.4%)	

### Descriptive analyses

Descriptive statistics and intercorrelations of all variables are presented in Table 1. In general, mean values for autonomy and its facets are at a medium (decision and design latitude, overall autonomy) to higher level (activity latitude).

The intercorrelations within the working conditions show that higher demands are also associated with higher latitudes. The intercorrelations within the health outcomes show the expected correlations; for example, higher exhaustion is associated with lower workability and lower well-being.

Correlations between working conditions and health outcomes show that autonomy, with the exception of cognitive irritation, has the expected associations with positive and negative health aspects, e.g. higher autonomy is associated with lower anxiety and higher workability.

### Results of regression analyses

A total of six moderated hierarchical multiple regressions were conducted to test for nonlinear relationships of autonomy and high cognitive demands on emotional exhaustion, cynicism, cognitive irritation, anxiety, well-being, and workability. The results of the hierarchical moderated regression analysis of autonomy are presented in the Tables 4, 5, 6, 7, 8, 9. The results of the regression analyses with the autonomy facets activity, design and decision latitude can be found in the appendix.

**Table 4:** Hierarchical moderated regression analyses predicting emotional exhaustion

	Step 1 (control variables)			Step 2 (linear effects)			Step 3 (quadratic effects)			Step 4 (cubic effects)		
	*B*	SE	*β*	*B*	SE	*β*	*B*	SE	*β*	*B*	SE	*β*
Control variable (step 1)												
Age	0.006	0.007	0.064	0.006	0.007	0.068	0.007	0.007	0.070	0.006	0.007	0.067
Main effects and linear two-way interaction (step 2)												
Autonomy				− 0.342	0.183	− 0.229 +	− 1.905	1.140	− 1.274 +	− 11.226	3.865	− 7.507**
Cognitive demands				0.859	0.768	0.333	1.038	2.815	0.402	7.429	3.758	2.881*
Autonomy * cognitive demands				− 0.113	0.238	− 0.164	0.117	1.729	0.170	− 3.756	2.294	− 5.450
Quadratic two-way interaction (step 3)												
Autonomy^2^							0.264	0.190	1.169	3.539	1.313	15.703**
Autonomy^2^ * cognitive demands							− 0.078	0.262	− 0.472	0.484	0.341	2.939
Cubic two-way interaction (step 4)												
Autonomy^3^										− 0.361	0.143	− 8.521*
Autonomy^3^ * cognitive demands												
*R*^2^			0.004			0.075			0.090			0.121
∆*R*			0.004			0.071**			0.015			0.031*
*F* (*df*)			0.753 (1,186)			3.685** (3,183)			2.969** (2,181)			3.528*** (1,180)

+ *p*-Wert ≤ .10 *p-Wert ≤ .05, **p-Wert ≤ .01, ***p-Wert ≤ .001 Cognitive Demands = Cluster of participants with high (1) and lower/medium (0) cognitive demands

**Table 5:** Hierarchical moderated regression analyses predicting cynicism

	Step 1 (control variables)			Step 2 (linear effects)			Step 3 (quadratic effects)			Step 4 (cubic effects)		
	*B*	SE	*β*	B	SE	*β*	B	SE	*β*	B	SE	*β*
Control variable (step 1)												
Age	− 0.008	0.007	− 0.083	− 0.007	0.007	− 0.077	− 0.007	0.007	− 0.078	− 0.008	0.007	− 0.081
Main effects and linear two-way interaction (step 2)												
Autonomy				− 0.381	0.188	− 0.252*	− 2.239	1.179	− 1.480 +	− 14.357	3.938	− 9.490***
Cognitive Demands				0.027	0.788	0.010	− 2.024	2.896	− 0.772	6.524	3.820	2.385
Autonomy * Cognitive Demands				0.061	0.244	0.087	1.645	1.781	2.349	− 3.368	2.333	− 4.809
Quadratic two-way interaction (step 3)												
Autonomy^2^							0.313	0.196	1.373	4.569	1.336	20.033***
Autonomy^2^ * Cognitive Demands							− 0.275	0.270	− 1.645	0.452	0.347	2.702
Cubic two-way interaction (step 4)												
Autonomy^3^										− 0.468	0.146	− 10.932**
Autonomy^3^ * cognitive demands												
*R*^2^			0.007			0.051			0.064			0.115
∆*R*			0.007			0.044*			0.013			0.051**
*F* (*df*)			1.287 (1,185)			2.425* (3,182)			2.054 + (2,180)			3.331** (1,179)

+ *p*-Wert ≤ .10 *p-Wert ≤ .05, **p-Wert ≤ .01, ***p-Wert ≤ .001. Cognitive Demands = Cluster of participants with high (1) and lower/medium (0) cognitive demands

**Table 6:** Hierarchical moderated regression analyses predicting anxiety

	Step 1 (control variables)			Step 2 (linear effects)			Step 3 (quadratic effects)			Step 4 (cubic effects)		
	*B*	SE	*β*	*B*	SE	*β*	*B*	SE	*β*	*B*	SE	*β*
Control variable (step 1)												
Age	− 0.005	0.007	− 0.055	− 0.003	0.006	− 0.035	− 0.003	0.006	− 0.032	− 0.003	0.006	− 0.034
Main effects and linear two-way interaction (step 2)												
Autonomy				− 0.265	0.171	− 0.186	0.423	1.063	0.298	− 6.787	3.625	− 4.781 +
Cognitive demands				0.616	0.716	0.251	4.774	2.627	1.958 +	9.724	3.528	3.967**
Autonomy * cognitive demands				− 0.248	0.222	− 0.379	− 2.799	1.614	− 4.274 +	− 5.799	2.154	− 8.856**
Quadratic two-way interaction (step 3)												
Autonomy^2^							− 0.116	0.177	− 0.542	2.418	1.232	11.300 +
Autonomy^2^ * cognitive demands							0.376	0.244	2.401	0.811	0.320	5.183*
Cubic two-way interaction (step 4)												
Autonomy^3^										− 0.279	0.134	− 6.942*
Autonomy^3^ * cognitive demands												
*R*^2^			0.003			0.113			0.126			0.147
∆*R*			0.003			0.110***			0.014			0.021*
*F* (*df*)			0.556 (1,185)			5.773*** (3,182)			4.336*** (2,180)			4.402*** (1,179)

+ *p*-Wert ≤ .10 *p-Wert ≤ .05, **p-Wert ≤ .01, ***p-Wert ≤ .001. Cognitive Demands = Cluster of participants with high (1) and lower/medium (0) cognitive demands

**Table 7:** Hierarchical moderated regression analyses predicting cognitive irritation

	Step 1 (control variables)			Step 2 (linear effects)			Step 3 (quadratic effects)			Step 4 (cubic effects)		
	B	SE	*β*	B	SE	*β*	B	SE	*β*	B	SE	*β*
Control variable (step 1)												
Age	0.022	0.009	0.174*	0.021	0.009	0.169*	0.022	0.009	0.173*	0.021	0.009	0.172*
Main effects and linear two-way interaction (step 2)												
Autonomy				− 0.341	0.241	− 0.172	− 1.932	1.480	− 0.975	− 10.332	5.060	− 5.212*
Cognitive demands				0.659	1.012	0.193	6.554	3.652	1.917 +	12.314	4.921	3.602*
Autonomy * cognitive demands				0.062	0.313	0.068	− 3.005	2.243	− 3.289	− 6.496	3.004	− 7.110*
Quadratic two-way interaction (step 3)												
Autonomy^2^							0.268	0.246	0.898	3.221	1.719	10.778 +
Autonomy^2^ * cognitive demands							0.381	0.339	1.745	0.888	0.446	4.063*
Cubic two-way interaction (step 4)												
Autonomy^3^										− 0.325	0.187	− 5.792 +
Autonomy^3^ * cognitive demands												
*R*^2^			0.030			0.085			0.128			0.142
∆*R*			0.030*			0.054*			0.043*			0.014 +
*F* (*df*)			5.840* (1,186)			4.243** (3,183)			4.423*** (2,181)			4.263*** (1,180)

+ *p*-Wert ≤ .10, *p-Wert ≤ .05, **p-Wert ≤ .01, ***p-Wert ≤ .001. Cognitive Demands = Cluster of participants with high (1) and lower/medium (0) cognitive demands

**Table 8:** Hierarchical moderated regression analyses predicting workability index

	Step 1 (control variables)			Step 2 (linear effects)			Step 3 (quadratic effects)			Step 4 (cubic effects)		
	*B*	SE	*β*	*B*	SE	*β*	*B*	SE	*β*	*B*	SE	β
Control variable (step 1)												
Age	− 0.002	0.004	− 0.036	− 0.003	0.004	− 0.048	− 0.003	0.004	− 0.049	− 0.003	0.004	− 0.048
Main effects and linear two-way interaction (step 2)												
Autonomy				0.279	0.101	0.333**	0.744	0.636	0.888	2.790	2.187	3.330
Cognitive demands				− 0.013	0.426	− 0.009	− 0.082	1.570	− 0.057	− 1.485	2.127	− 1.028
Autonomy * cognitive demands				− 0.018	0.132	− 0.048	− 0.078	0.964	− 0.202	0.772	1.299	2.000
Quadratic two-way interaction (step 3)												
Autonomy^2^							− 0.078	0.106	− 0.621	− 0.797	0.743	− 6.315
Autonomy^2^ * cognitive demands							0.022	0.146	0.237	− 0.101	0.193	− 1.099
Cubic two-way interaction (step 4)												
Autonomy^3^										0.079	0.081	3.339
Autonomy^3^ * cognitive demands												
*R*^2^			0.001			0.093			0.098			0.102
∆*R*			0.001			0.092***			0.004			0.005
*F* (*df*)			0.242 (1,186)			4.705*** (3,183)			3.261** (2,181)			2.931** (1,180)

+ *p*-Wert ≤ .10 *p-Wert ≤ .05, **p-Wert ≤ .01, ***p-Wert ≤ .001. Cognitive Demands = Cluster of participants with high (1) and lower/medium (0) cognitive demands

**Table 9:** Hierarchical moderated regression analyses predicting well-being

	Step 1 (control variables)			Step 2 (linear effects)			Step 3 (quadratic effects)			Step 4 (cubic effects)		
	*B*	SE	*β*	*B*	SE	*β*	*B*	SE	*β*	*B*	SE	*β*
Control variable (step 1)												
Age	0.012	0.007	0.136 +	0.011	0.006	0.123 +	0.011	0.006	0.122 +	0.011	0.006	0.123 +
Main effects and linear two-way interaction (step 2)												
Autonomy				0.418	0.175	0.291*	− 0.373	1.095	− 0.259	4.404	3.758	3.065
Cognitive demands				0.430	0.734	0.173	− 2.391	2.705	− 0.964	− 5.670	3.657	− 2.286
Autonomy * cognitive demands				− 0.106	0.227	− 0.160	1.693	1.661	2.555	3.681	2.233	5.554
Quadratic two-way interaction (step 3)												
Autonomy^2^							0.133	0.182	0.615	− 1.546	1.277	− 7.137
Autonomy^2^ * cognitiv							− 0.274	0.251	− 1.730	− 0.562	0.332	− 3.551
Cubic two-way interaction (step 4)												
Autonomy^3^										0.185	0.139	4.544
Autonomy^3^ * cognitive demands												
*R*^2^			0.018			0.090			0.096			0.105
∆*R*			0.018+			0.072**			0.006			0.009
*F* (*df*)			3.484+ (1,185)			4.508** (3,182)			3.191** (2,180)			2.999** (1,179)

+ *p*-Wert ≤ .10 *p-Wert ≤ .05, **p-Wert ≤ .01, ***p-Wert ≤ .001. Cognitive Demands = Cluster of participants with high (1) and lower/medium (0) cognitive demands

Overall, the models explain only a little of the variance in the sample. No consistently significant effects are found for the health variables. Anxiety is the health outcome that is most strongly associated with the modeled relations. For none of the health outcomes moderating effects of cognitive demands were found. The results in more detail:

### Autonomy and health outcomes

In the first step, the control variable age was tested. It is only for cognitive irritation and thus the first model significant ((*β* = 0.174, *p* < 0.05; *F* (1,186 = 5.840, *p* < 0.05). The older the employees are, the higher the reported cognitive irritation (see Table 7).

The linear effects of autonomy and cognitive demands as well as the linear interaction term were included in the second step. This second model is significant for all surveyed health parameters: emotional exhaustion (*F* (3,183) = 3.685, *p* < 0.01) (see Table 4), cynicism (*F* (3,182) = 2.425, *p* < 0.05) (see Table 5), and anxiety (*F* (3,182) = 5. 773, *p* < 0.001) (see Table 6), cognitive irritation (*F* (3,183) = 4.243, *p* < 0.01) (see Table 7), workability (*F* (3,183) = 4.705, *p* < 0.001) (see Table 8), and well-being (*F* (3,182) = 4.508, *p* < 0.01) (see Table 9). Cognitive demands as well as the interaction between cognitive demands and autonomy have no significant effect on any of the examined health variables. Linear associations of autonomy are found for cynicism (*β* = − 0.252, *p* < 0.05); that is, with increasing autonomy, cynicism decreases. Workability (*β* = − 0.333, *p* < 0.01) and well-being (*β* = 0.291, *p* < 0.05) are higher with higher autonomy.

Squared terms were included in the third step. The model was significant for cognitive irritation (*F *(2,181) = 4.423, *p* < 0.001), but no single variables were identified to account for the effect (see Table 7).

In the last step, the cubic terms were included. The fourth models were significant for emotional exhaustion (*F *(1,180) = 3.528, *p* < 0.001) (see Table 4), cynicism (*F *(1,179) = 3.331, *p* < 0.01) (see Table 5) and anxiety (*F *(1,179) = 4.402, *p* < 0.001) (see Table 6). The cubic autonomy term shows significant regression weights for emotional exhaustion (*β* = − 8.521, *p* < 0.05), cynicism (*β* = − 10.932, *p* < 0.01), and anxiety (*β* = − 6.942, *p* < 0.05).

### Facets of autonomy and health outcomes

#### Activity latitude

Using activity latitude as an independent variable, there were neither significant regression weights nor significant models for the health outcomes emotional exhaustion, cynicism, and well-being.

The first model is significant for cognitive irritation (*F* (1,186) = 5.840, *p* < 0.05) (see Appendix Table 13). The relation to the control variable age is significant (*β* = 0.174, *p* < 0.05).

Linear effects with activity latitude were only found for workability (*β* = 0.310, *p* < 0.01) (see Appendix Table 14). Quadratic effects were not found for any of the health outcomes. Cubic effects of activity latitude are only found for anxiety (*β* = − 8.525, *p* < 0.05). This fourth model is significant (*F* (1,179) = 2.819, *p* < 0.01) (see Appendix Table 12).

#### Design latitude

When design latitude is used as the independent variable, neither significant models nor significant regression weights are found for the outcomes emotional exhaustion, cynicism, workability, and cognitive irritation. In addition to the effect of the control variable age on cognitive irritation, linear effects with design latitude are only found for well-being (*β* = 0.334, *p* < 0.01); this second model is significant (*F *(3,182) = 5.217; *p* < 0.001) (see Appendix Table 21).

Analogous to activity latitude, there are no significant quadratic effects. Cubic effects with design latitude and the entire fourth model are significant for anxiety (*β* = − 5.277, *p* < 0.05; *F *(1,179) = 3.698; *p* < 0.001) (see Appendix Table 18).

#### Decision latitude

Using decision latitude as an independent variable, no significant models or regression weights are found for cynicism and well-being.

Linear effects with decision latitude and significant second models are found for emotional exhaustion (*β* = − 0.283, *p* < 0.05; (*F *(3,183) = 3.449; *p* < 0.05) (see Appendix Table 22), anxiety (*β* = − 0.276, *p* < 0.05; *F *(3,182) = 5.786; *p* < 0.001) (see Appendix Table 24), and workability (*β* = 0.356; *p* < 0.01; *F *(3,183) = 3.023; *p* < 0.05) (see Appendix Table 26).

Beyond that, however, neither quadratic nor cubic effects of decision latitude can be found between decision latitude and health outcomes.

## Discussion

The present study investigated whether the level of task-related cognitive demands moderates the influence of autonomy on employees' health. Autonomy was examined both as an overall construct and in its three dimensions of latitude to act, design, and decide. Task-related cognitive demands were operationalized with two clusters consisting of knowledge and mental demands as well as skill variety, information processing, problem solving, and complexity.

Our first hypothesis, a positive linear relationship between autonomy and various health outcomes such as emotional exhaustion, was confirmed, following the findings of many other studies (e.g., Van Rysselveldt et al., 2011). This result is evident in both the results of our correlation analysis and those of our regression analyses. Workability and well-being were the only two health outcomes to show purely linear correlations. Our data show that an increase in autonomy and its facets is consistent with an increase in workability and well-being. Workability is surveyed in the context of other work demands, health status, and employee resources and thus also covers the individual prerequisites for performance (Ilmarinen 2007). The findings on well-being mirror those of other studies in this context (e.g., Cheung et al. 2015).

We found no evidence for our second hypothesis, that the level of task-related cognitive demands moderates any positive effect found. It is true that our analysis with hierarchical moderated regression analyses showed curvilinear (cubic) effects of autonomy on individual health outcomes. However, the effects and trajectories found were similar to those of other studies (e.g., Stiglbauer 2017), and we did not detect moderation by the level of task-related cognitive demands in any model. Additionally, no significant main effects were found for task-related cognitive demands.

For employees with high task-related cognitive demands, we expected nonlinear relationships with autonomy and health. What was surprising for us was that indications of a nonlinear relationship were also found for low and medium cognitive demands. Our assumption that high task-related cognitive demands act as a kind of reinforcer "on top" and reverse a positive effect of autonomy must therefore be rejected. One reason for this could be that other mechanisms of impact are present when non-linear relationships emerge even without complementary task-related cognitive demands. To understand these results, Karasek's definition of job control can be used. In the present study, various groups of people with different skills and education were examined. Crucial for the effect of job control is the match of decision latitude and intellectual discretion (Karasek 1979).

Our third hypothesis was confirmed to the extent that we had divergent results for the individual facets. Overall, however, we did not find any moderating influence of task-related cognitive requirements. An increase in decision-making latitude consistently showed an increase in health. Especially the assumption that decision latitude with the potentially highest task-related cognitive demands would lead to a tendency to overstrain and health impairment could not be confirmed. Decision-making latitude offers employees wide-ranging opportunities, as they can set their own tasks and task objectives. Again, a reference to Karasek (1979) shows that the match between decision authority and skill level is essential. Such a fit significantly reduces the possibility of overdemanding freedom.

The facet of design latitude describes, that when performing activities, employees can both contribute and develop their own ideas as well as implement them. Contrary to our expectation, we found nonlinear associations with health outcomes for this facet. For instance, emotional exhaustion, cynicism and cognitive irritation as well as anxiety are health outcomes that do not necessarily improve with more design latitude. A great deal of design latitude can be associated with risk and disrupts routine processes (Dewett 2006; Zhou and Hoever 2014). A structural lack of regulations and guidelines makes designing mandatory and thus entails an expanded effort in the form of structuring and planning (Bredehöft et al. 2015). This can add up to increased demands for self-organization and many new work requirements (Allvin et al. 2011; Höge 2011; Sichler 2006; Kubicek et al. 2014), which are then reflected in the nonlinear effects on health parameters.

In addition to the reasons for our findings already presented, there is a possible more general reason that is particularly suitable to explain the more or less consistent curvilinear and missing moderator effects. The general level of reported task-related cognitive demands in the sample is, in line with other research on cognitive demands in the modern world of work (Meyer and Hünefeld 2018), mostly average to high (see Fig. 1). Although the differences between the used moderator groups is quite clear, it could be that the assumed mechanism of overtaxed cognition already starts far below the applied threshold. Accordingly, both groups studied would be subject to it. This could explain the curvilinear correlations without moderation. Since we wanted to make overarching statements about the relationship between autonomy and task-related cognitive demands, we broadly defined the health outcomes, and differential findings emerged in the various domains. For workability and well-being, there was a linear increase with increasing autonomy. Emotional exhaustion and cynicism, on the other hand, did not necessarily decrease with increasing autonomy. These types of results confirm the approach we have taken to analyze a wide range of health outcomes. It becomes clear that positive and negative outcomes are to be evaluated differently. Positive outcomes show positive correlations with autonomy, that is, a strengthening of health, but this is not the same as a decrease in negative outcomes, that is, an improvement in health impairment.

In the case of cognitive irritation and anxiety, tendencies for quadratic and cubic effects of design and activity latitude were found. One explanation could be that employees lack the skills or knowledge to be able to use the latitude (Sichler 2006), and this lack leads to poorer mental health. It is also possible that there is a lack of a structured framework, for example, in the form of organizational conditions or social support, in which they can meet the demands (Dettmers and Bredehöft, 2020; Egan et al. 2007). If employees themselves have to make new decisions about structures, procedures, and processes, this can lead to excessive demands (Egan et al. 2007). High autonomy in the form of shifting entrepreneurial responsibility to the employees themselves requires a psychologically demanding form of self-organization and self-optimization (Niehaus 2012; Sparks et al. 2001), which can result in cognitive irritation (Dettmers and Bredehöft 2020). The negative consequences of this are especially noticeable when mistakes happen or wrong decisions are made (Lehner et al. 2013). In this context, anxiety is an important health outcome that has received too little attention so far. Fear of being wrong or making the wrong decision is highly relevant in the subjectification and self-optimization tendencies prevalent in the new world of work. This corresponds exactly to the way the concept of autonomy is located in the individual by research and the world of work (for example, Sichler 2006; Moldaschl and Voß, 2002). This perception of autonomy as a requirement for the individual makes the use of health outcomes such as anxiety necessary.

### Limitations

First of all, our study has a cross-sectional design, that is, we did not collect longitudinal data that would allow us to draw conclusions about developments over time. For the measurement of the independent variable autonomy and the moderator task-related cognitive demands this should not pose a problem as both come into effect at the same time. However, effects on health need some time to develop and a second measurement time would have been preferable. Nevertheless, due to the long duration of employment the participants reported in the same SME, it could be argued that at least part of developments are captured in the data. Moreover, our results are not based on objective data but on self-reports. Due to common method bias observed relationships could be inflated or deflated depending on the question whether correlation between methods is higher or lower than the observed correlation without common methods (Podsakoff et al. 2003). Results show small to medium-sized correlations between the variable groups in question (autonomy, task-related cognitive demands and health outcomes) so that inflation probability is rather low. In addition, existing quadratic or interaction effects are deflated by common method variance (Siemsen et al. 2010) making them harder to detect. Work characteristics as well as cognitive demands are objective environmental factors but need to be internally processed. Again, it is difficult to collect these variables using objective methods, such as workplace observation. The collection of physiological health parameters could have been an enrichment for our results.

By clustering the different aspects of task-related cognitive demands, we succeeded in covering a broad range of possible influences and thus did not form an isolated moderator. However, we cannot exclude the possibility that other influences or requirements are relevant. Another limitation may be the sample per se. On the one hand, the response rate is on the low side of average response rates in applied contexts (Anseel et al. 2010; Baruch and Holtom 2008; Weigold et al. 2019) driven by factors in the companies we could not change. Nevertheless, sample size is sufficient for the performed analyses to find even small effects with a power of 0.90 (G*Power, Faul et al. 2009). On the other hand, due to the different response rates in the participating companies, sample attrition cannot be excluded. Although we were able to include three different companies and different occupational groups, it is not possible to generalize the results due to the small sample. In addition, the clustering of employees into those with high cognitive demands and those with lower cognitive demands shows a strong bias toward male study participants. On the other hand, the large proportion of men in the group with high cognitive demands is related to the still widespread social reality that men are more likely to hold positions and jobs that make such demands.

## Conclusions

Due to its positive effect on the health of employees, autonomy is an essential component of workplace design that promotes health and personality. In addition, this study illustrates that even well-known resources such as autonomy should always be considered in an organizational and social context.

## Data Availability

The datasets generated during and/or analysed during the current study are available from the corresponding author on reasonable request.

## Appendix

See Tables

**Table 10:** Hierarchical moderated regression analyses predicting emotional exhaustion

	*B*	SE	*β*	*B*	SE	*β*	*B*	SE	*β*	*B*	SE	*β*
Control variable (step 1)												
Age	0.006	0.007	0.064	0.005	0.007	0.051	0.004	0.007	0.047	0.004	0.007	0.043
Main effects and linear two-way interaction (step 2)												
Acitivity latitude				− 0.181	0.161	− 0.130	− 1.245	1.280	− 0.890	− 8.617	4.867	− 6.160 +
Cognitive demands				0.697	0.827	0.270	2.086	3.367	0.809	4.950	3.817	1.919
Acitivity latitude * cognitive demands				− 0.094	0.214	− 0.155	− 0.693	1.861	− 1.147	− 2.261	2.106	− 3.743
Quadratic two-way interaction (step 3)												
Acitivity latitude^2^							0.151	0.180	0.805	2.409	1.450	12.863 +
Acitivity latitude^2^ * cognitive demands							0.062	0.249	0.483	0.267	0.280	2.060
Cubic two-way interaction (step 4)												
Acitivity latitude^3^										− 0.219	0.140	− 6.877
Acitivity latitude^3^ * cognitive demands												
*R*^2^			0.004			0.038			0.049			0.062
∆*R*			0.004			0.034 +			0.012			0.013
*F* (*df*)			0.753 (1,186)			1.792 (3,183)			1.568 (2,181)			1.707 (1,180)

+ *p*-Wert ≤ .10 **p*-Wert ≤ .05, ***p*-Wert ≤ .01, ****p*-Wert ≤ .001. Cognitive Demands = Cluster of participants with high (1) and lower/medium (0) cognitive demands

10,

**Table 11:** Hierarchical moderated regression analyses predicting cynicism

	*B*	SE	*β*	*B*	SE	*β*	*B*	SE	*β*	*B*	SE	*β*
Control variable (step 1)												
Age	− 0.008	0.007	− 0.083	− 0.009	0.007	− 0.093	− 0.009	0.007	− 0.095	− 0.009	0.007	− 0.095
Main effects and linear two-way interaction (step 2)												
Acitivity latitude				− 0.247	0.163	− 0.174	− 1.073	1.310	− 0.759	− 0.916	4.993	− 0.648
Cognitive demands				0.273	0.837	0.104	0.359	3.430	0.137	0.297	3.931	0.113
Acitivity latitude * cognitive demands				− 0.040	0.217	− 0.065	0.024	1.897	0.039	0.058	2.171	0.095
Quadratic two-way interaction (step 3)												
Acitivity latitude^2^							0.117	0.184	0.618	0.069	1.490	0.364
Acitivity latitude^2^ * cognitive demands							− 0.019	0.254	− 0.146	− 0.024	0.289	− 0.180
Cubic two-way interaction (step 4)												
Acitivity latitude^3^										0.005	0.144	0.145
Acitivity latitude^3^ * cognitive demands												
*R*^2^			0.007			0.040			0.044			0.044
∆*R*			0.007			0.033			0.004			0.000
*F* (*df*)			1.287 (1,185)			1.901 (3,182)			1.378 (2,180)			1.175 (1,179)

+ *p*-Wert ≤ .10 **p*-Wert ≤ .05, ***p*-Wert ≤ .01, ****p*-Wert ≤ .001. Cognitive Demands = Cluster of participants with high (1) and lower/medium (0) cognitive demands

11,

**Table 12:** Hierarchical moderated regression analyses predicting anxiety

	*B*	SE	*β*	*B*	SE	*β*	*B*	SE	*β*	*B*	SE	*β*
Control variable (step 1)												
Age	− 0.005	0.007	− 0.055	− 0.005	0.006	− 0.052	− 0.005	0.006	− 0.055	− 0.005	0.006	− 0.060
Main effects and linear two-way interaction (step 2)												
Acitivity latitude				− 0.071	0.150	− 0.053	0.961	1.199	0.723	− 7.716	4.544	− 5.809 +
Cognitive demands				0.668	0.774	0.273	4.689	3.159	1.913	8.048	3.564	3.283*
Acitivity latitude * cognitive demands				− 0.266	0.200	− 0.463	− 2.544	1.746	− 4.433	− 4.383	1.966	− 7.637*
Quadratic two-way interaction (step 3)												
Acitivity latitude^2^							− 0.146	0.169	− 0.823	2.511	1.354	14.125 +
Acitivity latitude^2^ * cognitive demands							0.306	0.234	2.491	0.546	0.262	4.438*
Cubic two-way interaction (step 4)												
Acitivity latitude^3^										− 0.258	0.130	− 8.525*
Acitivity latitude^3^ * cognitive demands												
*R*^2^			0.003			0.071			0.080			0.099
∆*R*			0.003			0.068**			0.009			0.020*
*F* (*df*)			0.556 (1,185)			3.466** (3,182)			2.595* (2,180)			2.819** (1,179)

+ *p*-Wert ≤ .10 **p*-Wert ≤ .05, ***p*-Wert ≤ .01, ****p*-Wert ≤ .001. Cognitive Demands = Cluster of participants with high (1) and lower/medium (0) cognitive demands

12,

**Table 13:** Hierarchical moderated regression analyses predicting cognitive irritation

	*B*	SE	*β*	*B*	SE	*β*	*B*	SE	*β*	*B*	SE	*β*
Control variable (step 1)												
Age	0.021	0.009	0.174*	0.019	0.009	0.156*	0.019	0.009	0.155*	0.019	0.009	0.151*
Main effects and linear two-way interaction (step 2)												
Acitivity latitude				− 0.231	0.206	− 0.125	− 0.216	1.648	− 0.117	− 8.469	6.275	− 4.566
Cognitive demands				1.707	1.058	0.499	3.108	4.333	0.909	6.313	4.921	1.846
Acitivity latitude * cognitive demands				− 0.232	0.274	− 0.290	− 0.980	2.395	− 1.224	− 2.735	2.715	− 3.416
Quadratic two-way interaction (step 3)												
Acitivity latitude^2^							− 0.002	0.232	− 0.009	2.525	1.869	10.171
Acitivity latitude^2^ * cognitive demands							0.096	0.321	0.560	0.325	0.361	1.892
Cubic two-way interaction (step 4)												
Acitivity latitude^3^										− 0.245	0.180	− 5.806
Acitivity latitude^3^ * cognitive demands												
*R*^2^			0.030			0.103			0.104			0.113
∆*R*			0.030*			0.073**			0.001			0.009
*F* (*df*)			5.840* (1,186)			5.277*** (3,183)			3.513** (2,181)			3.291** (1,180)

+ *p*-Wert ≤ .10 **p*-Wert ≤ .05, ***p*-Wert ≤ .01, ****p*-Wert ≤ .001. Cognitive Demands = Cluster of participants with high (1) and lower/medium (0) cognitive demands

13,

**Table 14:** Hierarchical moderated regression analyses predicting Workability Index

	*B*	SE	*β*	*B*	SE	*β*	*B*	SE	*β*	*B*	SE	*β*
Control variable (step 1)												
Age	− 0.002	0.004	− 0.036	− 0.001	0.004	− 0.026	− 0.001	0.004	− 0.026	− 0.001	0.004	− 0.023
Main effects and linear two-way interaction (step 2)												
Acitivity latitude				0.243	0.089	0.310**	0.310	0.710	0.395	3.573	2.705	4.559
Cognitive demands				0.301	0.456	0.208	0.227	1.866	0.157	− 1.040	2.121	− 0.720
Acitivity latitude * cognitive demands				− 0.077	0.118	− 0.229	− 0.047	1.032	− 0.139	0.647	1.170	1.912
Quadratic two-way interaction (step 3)												
Acitivity latitude^2^							− 0.009	0.100	− 0.090	− 1.009	0.806	− 9.617
Acitivity latitude^2^ * cognitive demands							− 0.003	0.138	− 0.041	− 0.093	0.156	− 1.287
Cubic two-way interaction (step 4)												
Acitivity latitude^3^										0.097	0.078	5.433
Acitivity latitude^3^ * cognitive demands												
*R*^2^			0.001			0.069			0.069			0.077
∆*R*			0.001			0.068**			0.000			0.008
*F* (*df*)			0.242 (1,186)			3.404* (3,183)			2.249* (2,181)			2.157* (1,180)

+ *p*-Wert ≤ .10 **p*-Wert ≤ .05, ***p*-Wert ≤ .01, ****p*-Wert ≤ .001. Cognitive Demands = Cluster of participants with high (1) and lower/medium (0) cognitive demands

14,

**Table 15:** Hierarchical moderated regression analyses predicting well-being

	*B*	SE	*β*	*B*	SE	*β*	*B*	SE	*β*	*B*	SE	*β*
Control variable (step 1)												
Age	0.012	0.007	0.136 +	0.012	0.007	0.135 +	0.012	0.007	0.133 +	0.012	0.007	0.133 +
Main effects and linear two-way interaction (step 2)												
Acitivity latitude				0.186	0.154	0.138	− 1.291	1.227	− 0.960	− 2.130	4.698	− 1.584
Cognitive demands				0.436	0.791	0.176	− 1.058	3.231	− 0.426	− 0.733	3.684	− 0.296
Acitivity latitude * cognitive demands				− 0.046	0.205	− 0.079	0.945	1.786	1.627	0.767	2.033	1.321
Quadratic two-way interaction (step 3)												
Acitivity latitude^2^							0.209	0.173	1.163	0.466	1.399	2.592
Acitivity latitude^2^ * cognitive demands							− 0.147	0.239	− 1.182	− 0.124	0.271	− 0.996
Cubic two-way interaction (step 4)												
Acitivity latitude^3^										− 0.025	0.135	− 0.815
Acitivity latitude^3^ * cognitive demands												
*R*^2^			0.018			0.052			0.060			0.060
∆*R*			0.018 +			0.033			0.008			0.000
*F* (*df*)			3.484 + (1,185)			2.474* (3,182)			1.914 + (2,180)			1.637 (1,179)

+ *p*-Wert ≤ .10 **p*-Wert ≤ .05, ***p*-Wert ≤ .01, ****p*-Wert ≤ .001. Cognitive Demands = Cluster of participants with high (1) and lower/medium (0) cognitive demands

15,

**Table 16:** Hierarchical moderated regression analyses predicting emotional exhaustion

	*B*	SE	*β*	*B*	SE	*β*	*B*	SE	*β*	*B*	SE	*β*
Control variable (step 1)												
Age	0.006	0.007	0.064	0.007	0.007	0.073	0.007	0.007	0.070	0.006	0.007	0.068
Main effects and linear two-way interaction (step 2)												
Design latitude				− 0.201	0.136	− 0.183	− 1.303	0.658	− 1.182*	− 2.461	1.820	− 2.233
Cognitive demands				0.638	0.552	0.247	− 1.178	1.432	− 0.457	− 0.350	1.877	− 0.136
Design latitude * cognitive demands				− 0.069	0.176	− 0.103	1.392	0.965	2.080	0.847	1.254	1.266
Quadratic two-way interaction (step 3)												
Design latitude^2^							0.200	0.117	1.144 +	0.659	0.681	3.762
Design latitude^2^ * cognitive demands							− 0.253	0.155	− 1.698	− 0.171	0.197	− 1.146
Cubic two-way interaction (step 4)												
Design latitude^3^										− 0.054	0.079	− 1.620
Design latitude^3^ * cognitive demands												
*R*^2^			0.004			0.053			0.069			0.072
∆*R*			0.004			0.049*			0.016			0.002
*F* (*df*)			0.753 (1,186)			2.557* (3,183)			2.247* (2,181)			1.987 + (1,180)

+ *p*-Wert ≤ .10 **p*-Wert ≤ .05, ***p*-Wert ≤ .01, ****p*-Wert ≤ .001. Cognitive Demands = Cluster of participants with high (1) and lower/medium (0) cognitive demands

16,

**Table 17:** Hierarchical moderated regression analyses predicting cynicism

	*B*	SE	*β*	*B*	SE	*β*	*B*	SE	*β*	*B*	SE	*β*
Control variable (step 1)												
Age	− 0.008	0.007	− 0.083	− 0.007	0.007	− 0.073	− 0.007	0.007	− 0.076	− 0.008	0.007	− 0.081
Main effects and linear two-way interaction (step 2)												
Design latitude				− 0.227	0.140	− 0.203	− 0.897	0.680	− 0.803	− 3.890	1.878	− 3.481*
Cognitive demands				− 0.096	0.568	− 0.036	− 1.372	1.476	− 0.523	0.758	1.926	0.289
Design latitude * cognitive demands				0.078	0.180	0.115	1.077	0.996	1.586	− 0.318	1.284	− 0.468
Quadratic two-way interaction (step 3)												
Design latitude^2^							0.122	0.121	0.688	1.305	0.703	7.352 +
Design latitude^2^ * cognitive demands							− 0.170	0.161	− 1.127	0.040	0.202	0.263
Cubic two-way interaction (step 4)												
Design latitude^3^										− 0.138	0.081	− 4.121 +
Design latitude^3^ * cognitive demands												
*R*^2^			0.007			0.030			0.037			0.052
∆*R*			0.007			0.023			0.007			0.015 +
*F* (*df*)			1.287 (1,185)			1.406 (3,182)			1.137 (2,180)			1.402 (1,179)

+ *p*-Wert ≤ .10 **p*-Wert ≤ .05, ***p*-Wert ≤ .01, ****p*-Wert ≤ .001. Cognitive Demands = Cluster of participants with high (1) and lower/medium (0) cognitive demands

17,

**Table 18:** Hierarchical moderated regression analyses predicting anxiety

	*B*	SE	*β*	*B*	SE	*β*	*B*	SE	*β*	*B*	SE	*β*
Control variable (step 1)												
Age	− 0.005	0.00	− 0.055	− 0.00	0.006	− 0.031	− 0.002	0.006	− 0.023	− 0.002	0.006	− 0.028
Main effects and linear two-way interaction (step 2)												
Design latitude				− 0.172	0.127	− 0.165	− 0.091	0.616	− 0.087	− 3.674	1.681	− 3.511*
Cognitive demands				0.110	0.517	0.045	2.062	1.341	0.841	4.623	1.735	1.886**
Design latitude * cognitive demands				− 0.117	0.164	− 0.184	− 1.377	0.905	− 2.166	− 3.063	1.159	− 4.818**
Quadratic two-way interaction (step 3)												
Design latitude^2^							− 0.015	0.110	− 0.089	1.403	0.629	8.439*
Design latitude^2^ * cognitive demands							0.188	0.146	1.327	0.442	0.182	3.124*
Cubic two-way interaction (step 4)												
Design latitude^3^										− 0.166	0.073	− 5.277*
Design latitude^3^ * cognitive demands												
*R*^2^			0.003			0.085			0.101			0.126
∆*R*			0.003			0.082***			0.016			0.026*
*F* (*df*)			0.556 (1,185)			4.202** (3,182)			3.364** (2,180)			3.698*** (1,179)

+ *p*-Wert ≤ .10 **p*-Wert ≤ .05, ***p*-Wert ≤ .01, ****p*-Wert ≤ .001. Cognitive Demands = Cluster of participants with high (1) and lower/medium (0) cognitive demands

18,

**Table 19:** Hierarchical moderated regression analyses predicting cognitive irritation

	*B*	SE	*β*	*B*	SE	*β*	*B*	SE	*β*	*B*	SE	*β*
Control variable (step 1)												
Age	0.022	0.009	0.174*	0.021	0.009	0.172*	0.022	0.009	0.174*	0.022	0.009	0.174*
Main effects and linear two-way interaction (step 2)												
Design latitude				− 0.123	0.178	− 0.084	− 1.548	0.861	− 1.059 +	− 1.562	2.384	− 1.069
Cognitive demands				0.978	0.723	0.286	0.513	1.873	0.150	0.523	2.460	0.153
Design latitude * cognitive demands				− 0.067	0.230	− 0.075	0.631	1.263	0.711	0.624	1.643	0.704
Quadratic two-way interaction (step 3)												
Design latitude^2^							0.259	0.153	1.117 +	0.265	0.892	1.140
Design latitude^2^ * cognitive demands							− 0.152	0.203	− 0.772	− 0.151	0.258	− 0.767
Cubic two-way interaction (step 4)												
Design latitude^3^										− 0.001	0.103	− 0.015
Design latitude^3^ * cognitive demands												
*R*^2^			0.030			0.076			0.094			0.094
∆*R*			0.030*			0.046*			0.018			0.000
*F* (*df*)			5.840* (1,186)			3.763** (3,183)			3.112** (2,181)			2.653* (1,180)

+ *p*-Wert ≤ .10 **p*-Wert ≤ .05, ***p*-Wert ≤ .01, ****p*-Wert ≤ .001. Cognitive Demands = Cluster of participants with high (1) and lower/medium (0) cognitive demands

19,

**Table 20:** Hierarchical moderated regression analyses predicting workability index

	*B*	SE	*β*	*B*	SE	*β*	*B*	SE	*β*	*B*	SE	*β*
Control variable (step 1)												
Age	− 0.002	0.004	− 0.036	− 0.003	0.004	− 0.058	− 0.003	0.004	− 0.059	− 0.003	0.004	− 0.057
Main effects and linear two-way interaction (step 2)												
Design latitude				0.117	0.075	0.190	− 0.103	0.366	− 0.166	0.589	0.004	− 0.057
Cognitive demands				− 0.311	0.305	− 0.215	− 0.677	0.797	− 0.468	− 1.171	1.045	− 0.810
Design latitude * cognitive demands				0.094	0.097	0.250	0.387	0.537	1.033	0.712	0.698	1.901
Quadratic two-way interaction (step 3)												
Design latitude^2^							0.040	0.065	0.407	− 0.234	0.379	− 2.381
Design latitude^2^ * cognitive demands							− 0.051	0.087	− 0.608	− 0.100	0.110	− 1.196
Cubic two− way interaction (step 4)												
Design latitude^3^										0.032	0.044	1.726
Design latitude^3^ * cognitive demands												
*R*^2^			0.001			0.079			0.081			0.084
∆*R*			0.001			0.078**			0.002			0.003
*F* (*df*)			0.242 (1,186)			3.941** (3,183)			2.673* (2,181)			2.362* (1,180)

+ *p*-Wert ≤ .10 **p*-Wert ≤ .05, ***p*-Wert ≤ .01, ****p*-Wert ≤ .001. Cognitive Demands = Cluster of participants with high (1) and lower/medium (0) cognitive demands

20,

**Table 21:** Hierarchical moderated regression analyses predicting well-being

	*B*	SE	*β*	*B*	SE	*β*	*B*	SE	*β*	*B*	SE	*β*
Control variable (step 1)												
Age	0.012	0.007	0.136 +	0.010	0.006	0.114	0.010	0.006	0.112	0.010	0.006	0.113
Main effects and linear two-way interaction (step 2)												
Design latitude				0.354	0.128	0.334**	0.467	0.622	0.441	1.474	1.721	1.392
Cognitive demands				0.419	0.518	0.169	0.032	1.355	0.013	− 0.688	1.777	− 0.277
Design latitude * cognitive demands				− 0.112	0.165	− 0.174	0.102	0.914	0.159	0.576	1.187	0.896
Quadratic two-way interaction (step 3)												
Design latitude^2^							− 0.021	0.111	− 0.122	− 0.419	0.644	− 2.491
Design latitude^2^ * cognitive demands							− 0.027	0.147	− 0.191	− 0.099	0.186	− 0.690
Cubic two-way interaction (step 4)												
Design latitude^3^										0.047	0.074	1.466
Design latitude^3^ * cognitive demands												
*R*^2^			0.018			0.103			0.104			0.106
∆*R*			0.018 +			0.084***			0.001			0.002
*F* (*df*)			3.484 + (1,185)			5.217*** (3,182)			3.492** (2,180)			3.039** (1,179)

+ *p*-Wert ≤ .10 **p*-Wert ≤ .05, ***p*-Wert ≤ .01, ****p*-Wert ≤ .001. Cognitive Demands = Cluster of participants with high (1) and lower/medium (0) cognitive demands

21,

**Table 22:** Hierarchical moderated regression analyses predicting emotional exhaustion

	*B*	SE	*β*	*B*	SE	*β*	*B*	SE	*β*	*B*	SE	*β*
Control variable (step 1)												
Age	0.006	0.007	0.064	0.007	0.007	0.071	0.007	0.007	0.071	0.007	0.007	0.070
Main effects and linear two-way interaction (step 2)												
Decision latitude				− 0.368	0.171	− 0.283*	− 0.669	0.766	− 0.514	− 1.942	1.724	− 1.493
Cognitive demands				0.312	0.586	0.121	0.218	1.460	0.085	0.793	1.619	0.307
Decision latitude * cognitive demands				0.049	0.209	0.065	0.180	1.029	0.240	− 0.232	1.145	− 0.310
Quadratic two-way interaction (step 3)												
Decision latitude^2^							0.057	0.141	0.267	0.571	0.639	2.684
Decision latitude^2^ * cognitive demands							− 0.030	0.175	− 0.169	0.037	0.194	0.203
Cubic two-way interaction (step 4)												
Decision latitude^3^										− 0.062	0.075	− 1.495
Decision latitude^3^ * cognitive demands												
*R*^2^			0.004			0.070			0.071			0.075
∆*R*			0.004			0.066**			0.001			0.003
*F* (*df*)			0.753 (1,186)			3.449* (3,183)			2.315* (2,181)			2.078* (1,180)

+ *p*-Wert ≤ .10 **p*-Wert ≤ .05, ***p*-Wert ≤ .01, ****p*-Wert ≤ .001. Cognitive Demands = Cluster of participants with high (1) and lower/medium (0) cognitive demands

22,

**Table 23:** Hierarchical moderated regression analyses predicting cynicism

	*B*	SE	*β*	*B*	SE	*β*	*B*	SE	*β*	*B*	SE	*β*
Control variable (step 1)												
Age	− 0.008	0.007	− 0.083	− 0.007	0.007	− 0.075	− 0.008	0.007	− 0.079	− 0.008	0.007	− 0.081
Main effects and linear two-way interaction (step 2)												
Decision latitude				− 0.338	0.175	− 0.257 +	− 0.152	0.787	− 0.115	− 1.596	1.769	− 1.213
Cognitive demands				− 0.156	0.602	− 0.059	− 1.308	1.495	− 0.499	− 0.660	1.656	− 0.252
Decision latitude * cognitive demands				0.115	0.215	0.152	0.839	1.055	1.103	0.376	1.172	0.494
Quadratic two-way interaction (step 3)												
Decision latitude^2^							− 0.035	0.145	− 0.164	0.547	0.655	2.544
Decision latitude^2^ * cognitive demands							− 0.106	0.180	− 0.578	− 0.030	0.198	− 0.165
Cubic two-way interaction (step 4)												
Decision latitude^3^										− 0.070	0.077	− 1.673
Decision latitude^3^ * cognitive demands												
*R*^2^			0.007			0.043			0.053			0.057
∆*R*			0.007			0.036 +			0.009			0.004
*F* (*df*)			1.287 (1,185)			2.064 + (3,182)			1.675 (2,180)			1.553 (1,179)

+ *p*-Wert ≤ .10 **p*-Wert ≤ .05, ***p*-Wert ≤ .01, ****p*-Wert ≤ .001. Cognitive Demands = Cluster of participants with high (1) and lower/medium (0) cognitive demands

23,

**Table 24:** Hierarchical moderated regression analyses predicting anxiety

	*B*	SE	*β*	*B*	SE	*β*	*B*	SE	*β*	*B*	SE	*β*
Control variable (step 1)												
Age	− 0.005	0.007	− 0.055	− 0.003	0.006	− 0.031	− 0.003	0.006	− 0.030	− 0.002	0.006	− 0.028
Main effects and linear two-way interaction (step 2)												
Decision latitude				− 0.341	0.159	− 0.276*	0.476	0.710	0.385	2.643	1.591	2.140 +
Cognitive demands				− 0.122	0.545	− 0.050	0.578	1.353	0.236	− 0.401	1.494	− 0.164
Decision latitude * cognitive demands				− 0.032	0.195	− 0.046	− 0.683	0.954	− 0.960	0.020	1.057	0.028
Quadratic two-way interaction (step 3)												
Decision latitude^2^							− 0.154	0.131	− 0.763	− 1.029	0.590	− 5.095 +
Decision latitude^2^ * cognitive demands							0.128	0.163	0.750	0.014	0.179	0.081
Cubic two-way interaction (step 4)												
Decision latitude^3^										0.105	0.069	2.678
Decision latitude^3^ * cognitive demands												
*R*^2^			0.003			0.113			0.120			0.131
∆*R*			0.003			0.110***			0.007			0.011
*F* (*df*)			0.556 (1,185)			5.786*** (3,182)			4.090*** (2,180)			3.861*** (1,179)

+ *p*-Wert ≤ .10 **p*-Wert ≤ .05, ***p*-Wert ≤ .01, ****p*-Wert ≤ .001. Cognitive Demands = Cluster of participants with high (1) and lower/medium (0) cognitive demands

24,

**Table 25:** Hierarchical moderated regression analyses predicting cognitive irritation

	*B*	SE	*β*	*B*	SE	*β*	*B*	SE	*β*	*B*	SE	*β*
Control variable (step 1)												
Age	0.022	0.009	0.174*	0.020	0.009	0.163*	0.021	0.009	0.167*	0.021	0.009	0.164*
Main effects and linear two-way interaction (step 2)												
Decision latitude				− 0.427	0.225	− 0.248 +	0.372	1.006	0.215	− 2.543	2.253	− 1.474
Cognitive demands				− 0.538	0.771	− 0.157	1.469	1.915	0.430	2.785	2.116	0.814
Decision latitude * cognitive demands				0.474	0.275	0.477 +	− 1.042	1.350	− 1.049	− 1.985	1.496	− 1.999
Quadratic two-way interaction (step 3)												
Decision latitude^2^							− 0.151	0.185	− 0.535	1.025	0.835	3.637
Decision Latitude^2^ * cognitive demands							0.262	0.230	1.095	0.415	0.253	1.737
Cubic two-way interaction (step 4)												
Decision latitude^3^										− 0.142	0.098	− 2.579
Decision latitude^3^ * cognitive demands												
*R*^2^			0.030			0.084			0.090			0.101
∆*R*			0.030*			0.053*			0.007			0.010
*F* (*df*)			5.840* (1,186)			4.176** (3,183)			2.993** (2,181)			2.879** (1,180)

+ *p*-Wert ≤ .10 **p*-Wert ≤ .05, ***p*-Wert ≤ .01, ****p*-Wert ≤ .001. Cognitive Demands = Cluster of participants with high (1) and lower/medium (0) cognitive demands

25,

**Table 26:** Hierarchical moderated regression analyses predicting Workability Index

	*B*	SE	*β*	*B*	SE	*β*	*B*	SE	*β*	*B*	SE	*β*
Control variable (step 1)												
Age	− 0.002	0.004	− 0.036	− 0.002	0.004	− 0.047	− 0.002	0.004	− 0.042	− 0.002	0.004	− 0.043
Main effects and linear two-way interaction (step 2)												
Decision latitude				0.259	0.096	0.356**	0.756	0.429	1.037 +	0.539	0.967	0.740
Cognitive demands				0.367	0.329	0.254	1.349	0.817	0.934	1.447	0.908	1.001
Decision latitude * cognitive demands				− 0.137	0.118	− 0.325	− 0.902	0.576	− 2.150	− 0.972	0.642	− 2.317
Quadratic two-way interaction (step 3)												
Decision latitude^2^							− 0.094	0.079	− 0.787	− 0.006	0.358	− 0.052
Decision latitude^2^ * cognitive demands							0.135	0.098	1.340	0.147	0.109	1.453
Cubic two-way interaction (step 4)												
Decision latitude^3^										− 0.011	0.042	− 0.454
Decision latitude^3^ * cognitive demands												
*R*^2^			0.001			0.062			0.072			0.072
∆*R*			0.001			0.061**			0.010			0.000
*F* (*df*)			0.242 (1,186)			3.023* (3,183)			2.334* (2,181)			1.999 + (1,180)

+ *p*-Wert ≤ .10 **p*-Wert ≤ .05, ***p*-Wert ≤ .01, ****p*-Wert ≤ .001. Cognitive Demands = Cluster of participants with high (1) and lower/medium (0) cognitive demands

26,

**Table 27:** Hierarchical moderated regression analyses predicting well-being

	*B*	SE	*β*	*B*	SE	*β*	*B*	SE	*β*	*B*	SE	*β*
Control variable (step 1)												
Age	0.012	0.007	0.136 +	0.011	0.007	0.122 +	0.011	0.007	0.120 +	0.011	0.007	0.121 +
Main effects and linear two-way interaction (step 2)												
Decision latitude				0.311	0.164	0.249 +	− 0.036	0.738	− 0.029	0.012	1.665	0.009
Cognitive demands				0.504	0.565	0.203	− 0.012	1.406	− 0.005	− 0.034	1.562	− 0.014
Decision Latitude * Cognitive Demands				− 0.117	0.202	− 0.163	0.305	0.991	0.423	0.320	1.105	0.445
Quadratic two-way interaction (step 3)												
Decision latitude^2^							0.066	0.136	0.321	0.046	0.617	0.226
Decision latitude^2^ * cognitive demands							− 0.077	0.169	− 0.445	− 0.080	0.187	− 0.459
Cubic two-way interaction (step 4)												
Decision latitude^3^										0.002	0.073	0.059
Decision Latitude^3^ * Cognitive Demands												
*R*^2^			0.018			0.071			0.072			0.072
∆*R*			0.018 +			0.052*			0.001			0.000
*F* (*df*)			3.484 + (1,185)			3.468** (3,182)			2.331* (2,180)			1.987 + (1,179)

+ *p*-Wert ≤ .10 **p*-Wert ≤ .05, ***p*-Wert ≤ .01, ****p*-Wert ≤ .001. Cognitive Demands = Cluster of participants with high (1) and lower/medium (0) cognitive demands

27.
